# Behavioral Intention to Use a Smartphone Usage Management Application Between a Non-Problematic Smartphone Use Group and a Problematic Use Group

**DOI:** 10.3389/fpsyt.2021.571795

**Published:** 2021-06-17

**Authors:** Mun Joo Choi, Seo-Joon Lee, Sun Jung Lee, Mi Jung Rho, Dai-Jin Kim, In Young Choi

**Affiliations:** ^1^Department of Medical Informatics, College of Medicine, The Catholic University of Korea, Seoul, South Korea; ^2^Department of Biomedicine and Health Sciences, College of Medicine, The Catholic University of Korea, Seoul, South Korea; ^3^Department of Urology, College of Medicine, Seoul St. Mary's Hospital, The Catholic University of Korea, Seoul, South Korea; ^4^Department of Psychiatry, College of Medicine, Seoul St. Mary's Hospital, Addiction Research Institute, The Catholic University of Korea, Seoul, South Korea; ^5^Department of Psychiatry, College of Medicine, Seoul St. Mary's Hospital, The Catholic University of Korea, Seoul, South Korea; ^6^Catholic Institute for Healthcare Management and Graduate School of Healthcare Management and Policy, The Catholic University of Korea, Seoul, South Korea

**Keywords:** problematic smartphone use, smartphone usage management application, behavioral intention, TAM, ECT, MGCFA

## Abstract

Despite the many advantages of smartphone in daily life, there are significant concerns regarding their problematic use. Therefore, several smartphone usage management applications have been developed to prevent problematic smartphone use. The purpose of this study is to investigate the factors of users' behavioral intention to use smartphone usage management applications. Participants were divided into a smartphone use control group and a problematic use group to find significant intergroup path differences. The research model of this study is fundamentally based on the Technology Acceptance Model and Expectation-Confirmation Theory. Based on this theorem, models were modified to best suit the case of problematic smartphone use intervention by smartphone application. We conducted online surveys on 511 randomly selected smartphone users aged 20–60 in South Korea, in 2018. The Smartphone Addiction Proneness Scale was used to measure participants' smartphone dependency. Descriptive statistics were used for the demographic analysis and collected data were analyzed using IBM SPSS Statistics 24.0 and Amos 24.0. We found that in both non-problematic smartphone use group and problematic smartphone use group, facilitating factors and perceived security positively affect the intentions of users to use the application. One distinct difference between the groups was that the latter attributed a lower importance to perceived security than the former. Some of our highlighted unique points are envisioned to provide intensive insights for broadening knowledge about technology acceptance in the field of e-Addictology.

## Introduction

Smartphones have become crucial in everyday life worldwide, affecting all business, research, and social sectors (1, 2). Smartphone use is ever increasing, with usage in some countries reaching 90% and usage in most Western countries reaching more than half the population (3). Also, adults' smartphone usage reaches over 93% in South Korea (4). Despite the many advantages that smartphones provide to our daily lives, concerns related to problematic smartphone use have been increasing (3). Considering the advancement of Fourth Generation Mobile Communication Systems (4G) and Fifth Generation Mobile Communication Systems (5G) communication methods, smartphone usage is expected to increase even further.

The problem is that excessive smartphone use may lead to problematic smartphone use behavior. Problematic smartphone use is referred to as excessive smartphone use, which related with substance use disorder (5, 6). Meanwhile, according to ICD-11 or DSM-5, problematic smartphone use is yet defined as an addiction (7). Therefore, in this study, we use “Problematic Smartphone Use” instead of “Smartphone Addiction.” The term “problematic smartphone use” was used in a recent study of smartphone use types of psychiatric symptoms. According to a recent study by Chen et al., problematic smartphone use can be divided into two categories in the field of Internet addiction: general problematic smartphone use and specific problematic smartphone use (8–10). General problematic smartphone use indicates general behavioral patterns of excessive smartphone use, which may have negative consequences to the individuals (11). Specific problematic smartphone use indicates the use of smartphones that are problematic for certain types of smartphone activities (e.g., games, social networking service, etc.) (8–11). Prior research suggests that problematic smartphone use is associated with depression, anxiety, obsessive-compulsive behavior, and impulsiveness (12–14).

Internet of Things (IoT) is widely applied in many fields, most notably in healthcare (15, 16). Applied IoT in the medical field is called the Internet of Medical Things (IoMT) (17). The smartphone application (App) used in our research performs the function of IoMT, which makes it easier to collect and manage health data. In this regard, by adopting the IoMT, we were able to able to monitor the status of app users' continuous smartphone usage, and through collected data analysis and monitoring functions, it can be expected to be an effective solution for behavior change caused by the problematic smartphone use (18, 19).

In our previous research, we proposed the use of the Smartphone Overdependence Management System (SOMS), the smartphone background software app for collecting the usage data. This system was implemented to analyze the problematic smartphone use (20). Earlier researches using SOMS data were able to predict usage patterns that directly correlate with problematic smartphone use and classified problematic smartphone use with a data-driven prediction algorithm (21). According to this perspective, since SOMS functions well as an IoMT system, we have adopted SOMS as a smartphone usage management app. The app used in this study was enhanced by adding various factors that aim to prevent problematic smartphone use by providing personalized health care services based on the SOMS functions. The system was unique compared to other management systems which lacked a proper automated measurement algorithm (22). The idea of this study was to support behavior change in such a way that problematic smartphone use is controlled using smartphone technology, which has been widely and successfully applied to other healthcare systems (19).

The research model of this paper was fundamentally based on the Technology Acceptance Model (TAM) and Expectation-Confirmation Theory (ECT). The TAM is developed by Davis (23), which is a widely accepted and influential model that predicts users' perceptions or acceptance of information system use (24–26). The ECT was originally used for studying consumer satisfaction, post-purchase behavior, and service marketing in general (27), but its predictive ability has been demonstrated over a wide range of fields (27–29).

Based on the aforementioned background, the purpose of this study was to examine the factors that positively or negatively affect behavioral intention to use a system, in order to successfully develop an application and implement programs for users. We also aimed to find out the differences in the factors influencing the intention to use such a smartphone usage control system between those who have a general usage behavior and those with problematic usage behavior. For that, we divide them into non-problematic smartphone use group and problematic smartphone use group. The results will be compared with other related research regarding the behavioral intention to use smartphone devices and envisioned to be used as baseline data to increase the success rate when developing intervention programs using smartphone apps.

## Materials and Methods

### Study Design

**Figure 1:**
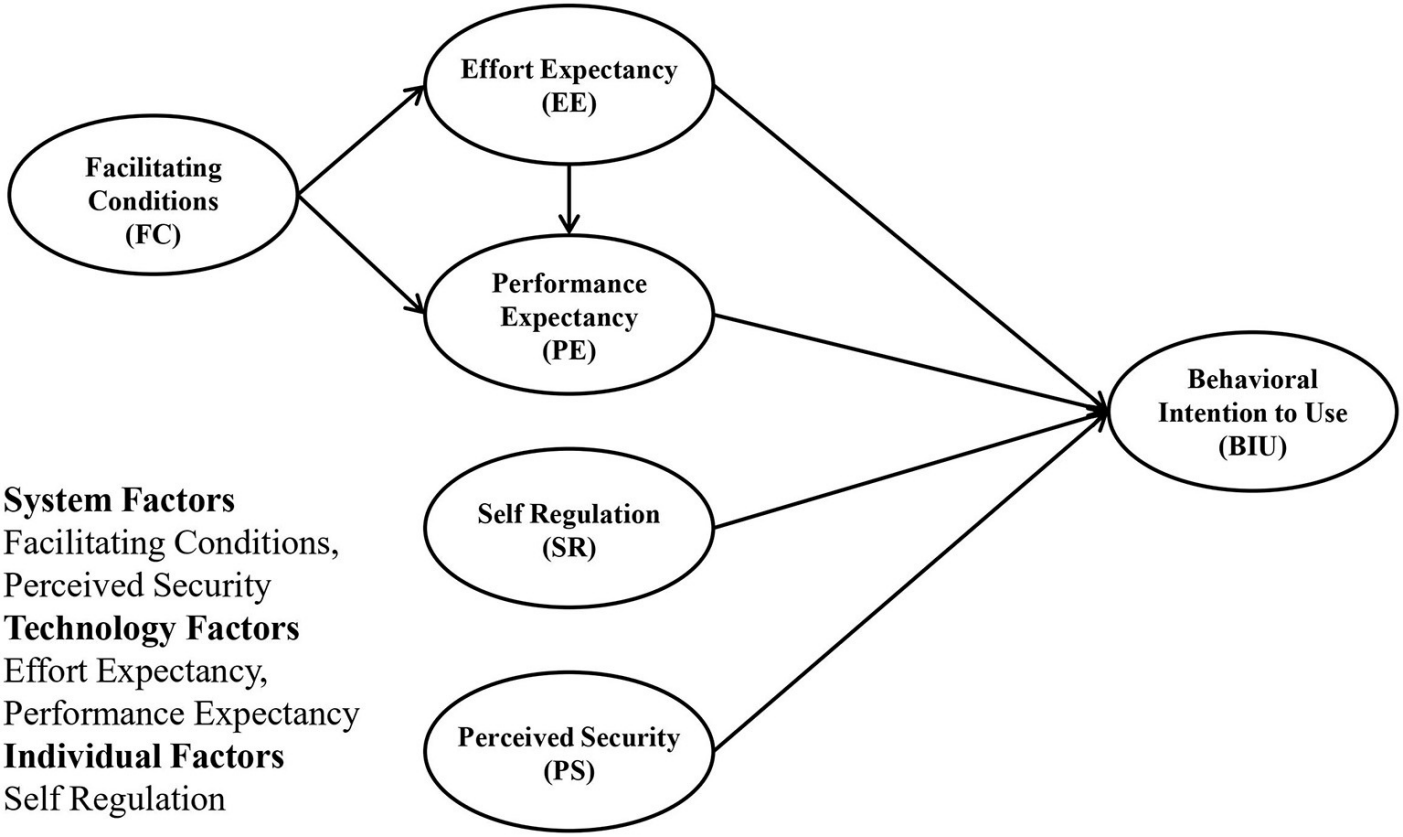
Modified study model.

Based on the fundamentals of TAM and ECT, we modified these models by converging, excluding, or including important variables that were identified as appropriate in the case of problematic smartphone use intervention by smartphone app, as shown in Figure 1. Because the main dynamics of TAM and ECT was similar, they were converged into the dynamic relation between facilitating conditions, effort expectancy, performance expectancy, and behavioral intention to use. In this model, perceived security was added, since personal security issues in network services have been a threat to many services, including in the field of healthcare, which obtains sensitive private information. Another factor, self-regulation, was added, because this was considered an important construct regarding problematic smartphone use behavior.

In this model, FC and PS comprise system factors, which are the factors that help facilitate information system use. Additionally, EE and PE represent technology factors that affect intention to use. Lastly, SR represents individual factors related to intention to use.

Research hypotheses have been tested in relation to the model proposed above in two groups and are shown as follows.

H1: FC has a significant influence on EE regarding intention to use smartphone usage management application in two groups.

H2: FC has a significant influence on PE regarding intention to use smartphone usage management application in two groups.

H3: EE has a significant influence on PE regarding intention to use smartphone usage management application in two groups.

H4: EE has a significant influence on BIU regarding intention to use smartphone usage management application in two groups.

H5: PE has a significant influence on BIU regarding intention to use smartphone usage management application in two groups.

H6: SR has a significant influence on BIU regarding intention to use smartphone usage management application in two groups.

H7: PS has a significant influence on BIU regarding intention to use smartphone usage management application in two groups.

As a pilot study to validate the questionnaire, confirmatory factor analysis was performed to observe how well the prior conceptualized, theoretically grounded model are constructed (related results are provided in Supplementary Materials).

### Study Subjects and Data Collection

**Figure 2:**
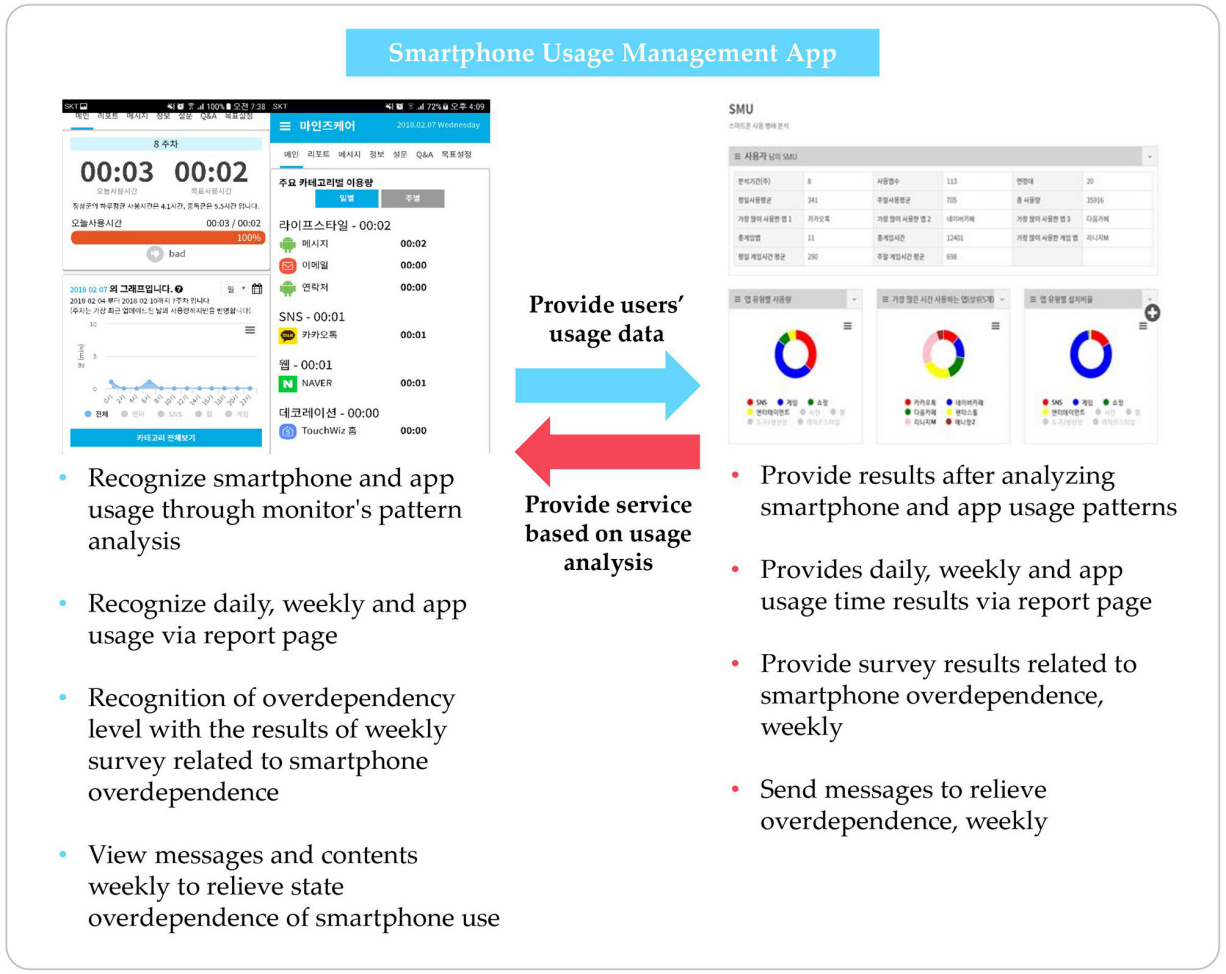
Description of developed application.

The online surveys were conducted anonymously from a social survey institution panel. Five hundred eleven smartphone users were randomly selected, who were of age 20 years or older. Participants were evenly pooled from metropolitan areas of South Korea in September 18–28, 2018. In this study, non-probability sampling methods were used. The survey were provided in Korean version (translated version available in Supplementary Materials). Only participants who used smartphones for at least 1 h per day were included in the study. Informed consent was obtained prior to the survey. Non-adult participants were excluded as parental consent is a legal requirement for underage research, and the approval process in the Korean Institutional Review Board is strict and difficult. Before the survey, participants were informed about the developed smartphone usage management app, as shown in Figure 2.

The size of the sample population was selected based on the following criteria. According to the March 2018 statistics, eight out of 10 people use smartphones (population *n* = 51,779,892) (4). For reliability within the 95% confidence interval, the appropriate recommended sample size was 480, but we successfully collected over 500 (30).

The study procedures were performed in accordance with the Declaration of Helsinki. The Institutional Review Board of the Catholic University of South Korea, St. Mary's Hospital (MC18QESI0065), approved the study.

### Measures

The Korean Smartphone Addiction Proneness Scale for Adults (S-Scale) was used for the two groups: non-problematic smartphone use (NPSU) group and problematic smartphone use (PSU) group. The S-Scale is a 15-item scale, rated on a four-point Likert scale ranging from “Strongly disagree” to “Strongly agree” from Kim et al., which measures smartphone addiction proneness scale for youth and adults (31, 32). The S-Scale is classified into three groups: high-risk (cutoff: ≥45), at-risk (44 ≥ x ≥ 42), and normal (41 ≥ x ≥0). In this study, we regrouped the high-risk and at-risk groups as the PSU group and the normal group as the NPSU group for convenience of analysis.

Facilitating conditions (FC) were defined by Venkatesh (33) as a factor that helps facilitate system use (34). A total of five questions was asked, rated on a five-point Likert scale ranging from “Strongly disagree” to “Strongly agree” with a higher score indicating a higher intention to use. In this study, the reliability of this measure is Cronbach's alpha 0.85.

Effort expectancy (EE) refers to how easy and comfortable a system is to use. This measure was defined by Davis (23) and Venkatesh and Davis (34), which comprises a total of five questions rated on a five-point Likert scale ranging from “Strongly disagree” to “Strongly agree” with a higher score indicating a higher intention to use (23, 33). In this study, the reliability of this measure is Cronbach's alpha 0.86. We deleted two items to improve internal reliability.

Performance expectancy (PE) represents how useful a system is for the PSU group. This measure was also defined by Davis (23) and Venkatesh and Davis (34), which includes a total of five questions measured on a five-point Likert scale, ranging from “Strongly disagree” to “Strongly agree” with a higher score indicating a higher intention to use (23, 33). In this study, the reliability of this measure is Cronbach's alpha 0.90.

Self-regulation (SR) is the scale of people's ability to control themselves. It was defined by Diehl, Semegon, and Schwarzer (35), which comprises a total of nine questions rated on a four-point Likert scale, ranging from “Strongly disagree” to “Strongly agree” with a higher score indicating a higher intention to use. In this study, the reliability of this measure is Cronbach's alpha 0.83. We deleted two items to improve internal reliability.

Perceived security (PS) measure was defined by David et al. (36) which comprises a total of five questions rated on a five-point Likert scale, ranging from “Strongly disagree” to “Strongly agree” with a higher score indicating a higher intention to use. In this study, the reliability of this measure is Cronbach's alpha 0.92.

Behavioral intention-to-use (BIU) measure was defined by Davis and Gefen et al. (23, 37) which comprises a total of three questions rated on a five-point Likert scale ranging from “Strongly disagree” to “Strongly agree” with a higher score indicating a higher intention to use. In this study, the reliability of this measure is Cronbach's alpha 0.88.

### Data Analysis

The collected data were analyzed using IBM SPSS Statistics 24.0 and Amos 24.0. Socio-demographic characteristics and the frequency and percentage of measurement variables were analyzed with descriptive statistics, and *t*-test was used to analyze differences between variables among the groups. Pearson's correlation coefficient was used to determine the correlation between variables. This study comprised a multigroup path analysis study to identify factors affecting the intention to use smartphone usage management app through FC, EE, PE, SR, PS, and BIU. Amos 24.0 was used to analyze the path difference between groups. The following procedure was conducted for analysis. First, we found the correlation between FC, EE, PE, SR, PS, and BIU, which are the main variables. Second, we constructed the hypothesized path model and measurement equivalence to determine whether both groups are recognized as the main variables identically, through multigroup confirmatory factor analysis. Third, through verification of the conducted path model, we found differences between groups on the direct effects of variables affecting intention to use smartphone usage management apps. To evaluate the goodness-of-fit index (GFI) of the research model, we used χ^2^ statistic, chi-square ratio χ^2^/df, Tucker–Lewis index (TLI), the comparative fit index (CFI), and the root mean square error of approximation (RMSEA) (38–40). The most basic measure of overall goodness of fit for evaluating the research model is the χ^2^ statistic, which is calculated based on the normal distribution of data and is sensitive to the size of the data. A good fit was obtained when the χ^2^/df value was ≤ 3 and the CFI value was >0.90. The smaller the RMSEA value is, the better the overall goodness of fit is. In general, <0.05 indicates very good fit, <0.08 indicates good fit, <0.10 indicates a normal fit, and above 0.10 indicates a poor fit. Furthermore, TLI and GFI values more than 0.90 indicate a good fit. However, acceptable RMSEA, CFI, or χ^2^/df values were enough to indicate goodness of fit, despite TLI and GFI values below 0.9 (41, 42). Multigroup confirmatory factor analysis is an analysis conducted before multigroup path analysis in order to find whether each group equally identifies the measurement survey items. The measurement invariance test was approached in five steps: (1) unconstrained, (2) measurement weights, (3) structural covariances, (4) structural covariances, and (5) measurement residuals (43). If the difference between the χ^2^ value of the unconstrained model and the χ^2^ of each constrained model is significant, this implies a significant difference between the groups. To confirm if PSU and NPSU perceived the variables identically, measurement equivalence was conducted through multigroup confirmatory factor analysis. The maximum likelihood estimation was used to estimate the model and analyze the *p*-value using a bootstrapping procedure to verify the significance of each path coefficient and indirect effect.

## Results

### Socio-Demographic Data and Correlations of Measured Variables

The socio-demographic results are shown in Table 1. A percentage of 64.1% (*N* = 328) of participants were NPSU and 35.8% (*N* = 183) were PSU. Our subjects ranged from age 20–50, with a relatively equal proportion for each age group. Most of our subjects were married (45.8%), were graduate school students (70.3%), and had white-collar occupations (41.9%). A vast proportion of our respondents (75.1%) did not experience using any smartphone usage management app. The most used apps were SNS (30.3%), followed by web surfing (26%), life style (11.4%), and game (10.6%).

**Table 1:** Characteristic of socio-demographics.

**Characteristics**		**NPSU (*N* = 328) (%)**	**PSU (*N* = 183) (%)**	**Overall (*N* = 511) (%)**	***χ^2^***	***p***
Gender	Male	164 (50.0)	85 (46.4)	249 (48.7)	0.593	0.441
	Female	164 (50.0)	98 (53.6)	262 (51.3)		
Age group	20–29	82 (25.0)	50 (27.3)	132 (25.8)	7.103	0.069
	30–39	76 (23.2)	54 (29.5)	130 (25.4)		
	40–49	82 (25.0)	48 (26.2)	130 (25.4)		
	Over 50	88 (26.8)	31 (16.9)	119 (23.3)		
Marital status	Married	145 (44.2)	89 (48.6)	234 (45.8)	1.804	0.406
	Unmarried	183 (55.8)	94 (51.4)	277 (54.3)		
Education	High school or lower	44 (13.4)	20 (10.9)	64 (12.5)	2.147	0.342
	College student	51 (15.5)	37 (20.2)	88 (17.2)		
	Graduate or above	233 (71.0)	126 (68.9)	359 (70.3)		
Occupation	White-collar	142 (43.3)	72 (39.3)	214 (41.9)	8.589	0.476
	Student	31 (9.5)	22 (12.0)	53 (10.4)		
	Professional	24 (7.3)	18 (9.8)	42 (8.2)		
	Unemployed	25 (8.2)	15 (8.2)	40 (7.8)		
	Others	106 (31.7)	56 (30.7)	162 (31.7)		
Experience to use smartphone usage management app	Yes	57 (17.4)	70 (38.3)	127 (24.9)	27.403	0.000
	No	271 (82.6)	113 (61.7)	384 (75.1)		
Playing smartphone game	Yes	158 (48.2)	124 (67.8)	282 (55.2)	18.225	0.000
	No	170 (51.8)	59 (32.2)	229 (44.8)		
Most used App for the past 1 year	SNS	94 (28.7)	61 (33.3)	155 (30.3)	15.171	0.297
	Web surfing	85 (25.9)	48 (26.2)	133 (26.0)		
	Game	32 (9.8)	22 (12.0)	54 (10.6)		
	Entertainment	30 (9.1)	12 (6.6)	42 (8.2)		
	Shopping	14 (4.3)	12 (6.6)	26 (5.1)		
	Lifestyle	47 (14.3)	11 (6.0)	58 (11.4)		
	Others	26 (7.9)	17 (9.3)	43 (8.5)		
Total		328	183	511		

*NPSU, Non-problematic smartphone use; PSU, problematic smartphone use*.

### Correlations of Measured Variables

The measurement models' fit indices, including the acceptable thresholds, are shown in Table 2.

**Table 2:** Goodness-of-fit statistics.

**Model-fit index**	**Recommended value**	**Scores**
Chi-square/degree of freedom (χ^2^/df)	≤ 3.00	2.370
Goodness-of-fit index (GFI)	≥0.90	0.890
Tucker–Lewis index (TLI)	≥0.90	0.892
Comparative fit index (CFI)	≥0.90	0.945
Root mean square error of approximation (RMSEA)	<0.1	0.052

The chi-square/degrees of freedom (χ^2^/df) was 2.370, the GFI was 0.890, the TLI was 0.892, the CFI was 0.945, and the RMSEA was 0.052. Although the values of the GFI and TLI were slightly lesser than recommended, it was concluded that all fit indices were acceptable and supported a reasonable fit assumption (44, 45). This was also supported by prior studies, which accepted models that had GFI or TLI values marginally lower, but with good fit RMSEA, CFI, or χ^2^/df value supplementing the lack of GFI or TLI (41, 42).

The results of analyzing the mean, standard deviation, and correlation of the variables are shown in Table 3. There was a positive correlation between FC and EE as well as in EE and PE. Additionally, there was a quantitative correlation between PE and SR, SR and PS, and PS and BIU.

**Table 3:** Correlations, means, and standard deviations for measured variables.

	**FC**	**EE**	**PE**	**SR**	**PS**	**BIU**
FC	1					
EE	0.531^**^	1				
PE	0.572^**^	0.623^**^	1			
SR	0.248^**^	0.252^**^	0.205^**^	1		
PS	0.263^**^	0.373^**^	0.496^**^	0.281^**^	1	
BIU	0.341^**^	0.430^**^	0.584^**^	0.184^**^	0.590^**^	1
Mean	3.649	3.531	3.373	2.578	2.874	2.836
SD	0.645	0.714	0.753	0.537	0.864	0.919

** *p < 0.01*.

*SD, Standard deviations; FC, facilitating conditions; EE, effort expectancy; PE, performance expectancy; SR, self-regulation; PS, perceived security; BIU, behavioral intention to use*.

### Multigroup Confirmatory Factor Analysis

The focus of this study was to determine the differences of intention to use smartphone usage management apps between groups. The results of this study confirmed that configure invariance was normal (unconstrained models fit χ^2^ = 1346.676, *p* < 0.001, TLI = 0.922, CFI = 0.931, RMSEA = 0.041). As a result of the χ^2^ test of the unconstrained model and constrained model 1, it was insignificant at the *p* < 0.05 level. Therefore, we were able to conduct the multigroup path analysis, since both of the groups' model form and measurement equivalence of factor coefficients were confirmed between latent and measured variables. As prior studies suggest that the chi-squared test was not suitable for the model-fit index, we were able to conduct multi-path analysis as other model-fit indexes (TLI, CFI, RMSEA) between the two groups were shown to be a good fit (Table 4) (46, 47).

**Table 4:** Multigroup confirmatory factor analysis.

**Model**	***χ^2^***	**Df**	**TLI**	**CFI**	**RMSEA**	***χ^2^* difference**	**df difference**	***p***
Unconstrained	1346.676	724	0.922	0.931	0.041			
Constrained 1^a^	1363.216	747	0.925	0.931	0.040	48.514	23	0.831
Constrained 2^b^	1389.133	745	0.922	0.928	0.041	67.272	21	0.004
Constrained 3^c^	1493.291	797	0.921	0.922	0.041	434.003	73	0.000
Constrained 4^d^	1601.674	826	0.915	0.914	0.043	317.004	102	0.000

a *Constrained 1: measurement weights*.

b *Constrained 2: structural covariances*.

c *Constrained 3: structural covariances*.

d *Constrained 4: measurement residuals*.

TLI, Tucker–Lewis Index; CFI, comparative fit index; RMSEA, root mean square error of approximation.

### Multigroup Path Analysis

**Figure 3:**
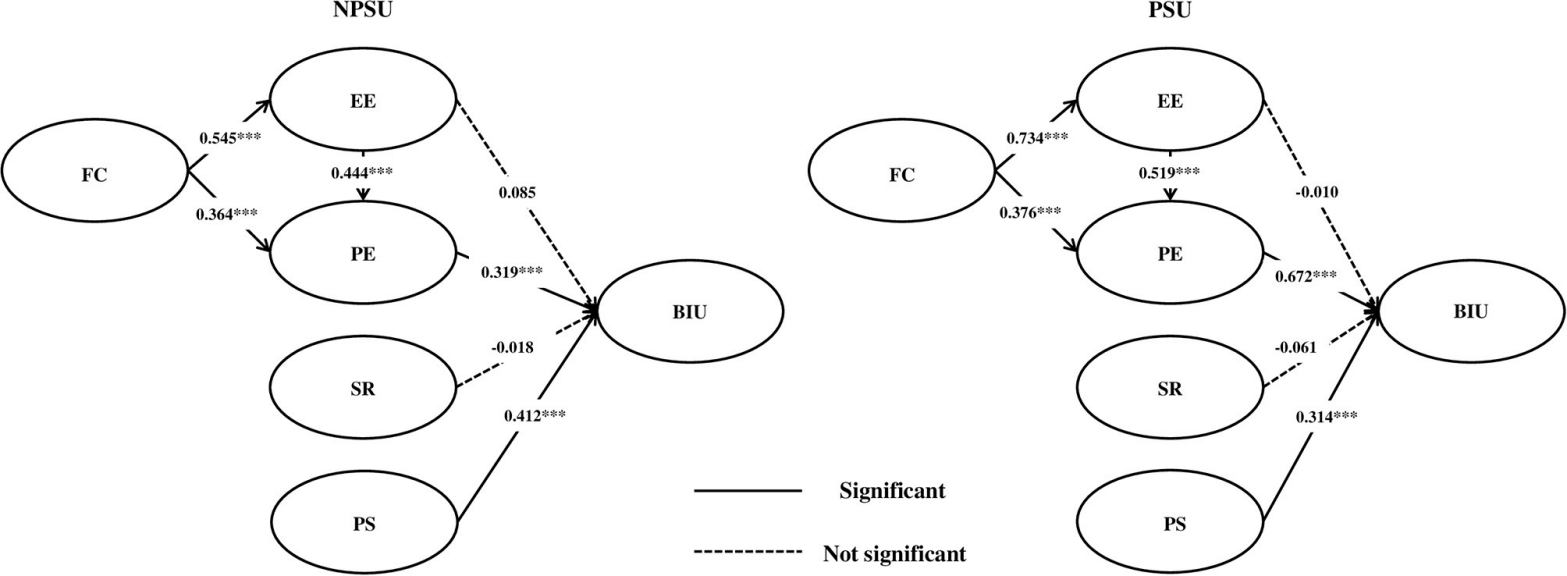
Result of multigroup path analysis.

The critical ratio (CR) value was also used to check whether the difference between groups was significant (intergroup path difference). As a result of this study, FC in the NPSU group had a significant positive effect on EE (β = 0.545, *p* < 0.001). In addition, the FC of the PSU group had a significant positive effect on EE (β = 0.734, *p* < 0.001). The difference in the FC→ EE pathway between groups was not statistically significant. FC in the NPSU group had a significant positive effect on PE (β = 0.364, *p* < 0.001). In addition, FC in the PSU group was found to have a significant effect on PE (β = 0.376, *p* < 0.001). The differences in the FC→ PE pathways between groups were not statistically significant. EE in the NPSU group was found to have a significant positive effect on PE (β = 0.444, *p* < 0.001). The EE of the PSU group was found to have a positive effect on PE (β = 0.519, *p* < 0.001). Differences in the EE→ PE pathway between groups were not statistically significant. In both NPSU and PSU, SR did not significantly affect BIU. PS in the NPSU group was found to have a significant effect on BIU (β = 0.412, *p* < 0.001). PS in the PSU group was found to have a significant effect on BIU (β = 0.314, *p* < 0.001). Differences in the PS→ BIU pathway among the groups were statistically significant (CR = 2.411 > 1.96). Both NPSU and PSU showed that EE had no significant effect on BIU. PE in the NPSU group had a significant positive effect on BIU (β = 0.319, *p* < 0.001). In addition, the PE of the PSU group was found to have a significant effect on BIU (β = 0.672, *p* < 0.001). The differences in the PE→ BIU pathways between groups were not statistically significant (Table 5, Figure 3).

**Table 5:** Multigroup path analysis.

**Path**	**NPSU**			**PSU**			**Intergroup path difference (C.R.)**
	**B**	**β**	**S.E**.	**B**	**β**	**S.E**.	
FC→ EE	0.509^***^	0.545^***^	0.059	0.705^***^	0.734^***^	0.082	1.950
FC→ PE	0.367^***^	0.364^***^	0.063	0.333^***^	0.376^***^	0.085	−0.319
EE→ PE	0.479^***^	0.444^***^	0.070	0.479^***^	0.519^***^	0.093	0.002
SR→ BIU	−0.041	−0.018	0.122	−0.119	−0.061	0.134	−0.793
PS→ BIU	0.447^***^	0.412^***^	0.058	0.297^***^	0.314^***^	0.067	2.411^*^
EE→ BIU	0.118	0.085	0.100	−0.012	−0.010	0.129	−0.430
PE→ BIU	0.411^***^	0.319^***^	0.092	0.837^***^	0.672^***^	0.151	−1.695

* *p < 0.05;*

*** *p < 0.001*.

*S.E., standard errors; C.R., Cretial ratio; NPSU, non-problematic smartphone use; PSU, problematic smartphone use; FC, facilitating conditions; EE, effort expectancy; PE, performance expectancy; SR, self-regulation; PS, perceived security; BIU, behavioral intention to use*.

A higher value of the coefficient means stronger intention to the relative variable. In this study, there was a statistically significant difference in the path of intergroup differences between PS→ BIU. This means that the PSU group considered the security factor less important than the NPSU group, and the difference between the two groups was significant.

## Discussion

### Common results for both NPSU and PSU

This section explains the common results found in both NPSU and PSU. FC→ EE was analyzed to prove that if certain facilitating conditions were met, it would significantly affect users by making them feel that less effort was needed to use the proposed monitoring system. Once users' expected effort was reduced, we predicted that it would have a positive effect on their intention to use EE→ BIU.

Although FC→ EE was significant, EE→ BIU all turned out to be statistically insignificant, contrary to many prior studies. For instance, a similar research by Bhattacherjee (48) suggested that users' continuance intention is determined by their satisfaction with information system use, which is positively affected by the expectation confirmation. Our results were inconsistent with another prior study (49), which reported that perceived ease of use as a similar variable to EE was a statistically significant determinant of BIU. Even psychologically, Melas et al. stated that users are naturally attracted to easy tools (25) rather than complex ones. Considering that even up-to-date research on subjects in Malaysia shows that easy usage leads to enhanced usability (50), our results may have been due to South Koreans' unique characteristics, with over 90% of the population already accustomed to smartphones. That is, EE may not be a significant factor for Koreans, who naturally take it for granted that use of a smartphone app is easy. This is a unique point of our research, considering that no studies have determined this relationship in South Korea to date.

Another unique and important point that should be noted here is that although the findings of EE→ BIU did not have a direct effect, nonetheless EE did have an indirect effect on BIU. That is, EE positively affected PE, and PE eventually positively affected BIU (this pathway will be discussed separately below). This link from FC→ EE→ PE and eventually to BIU was found to be statistically significant for both groups in our analysis. The core link from EE to PE enabled this phenomenon. Similarly, research conducted by Dhiman et al. (51) supported our link by investigating consumer adoption of smartphone fitness apps and revealing a significant relationship between EE and performance expectation.

An important point to note is that the finding of EE→ PE directly contradicts the findings of our 2018 research (49), in which we found that perceived ease of use had a statistically significant negative effect on BIU. It can be assumed that the more recent results may have been different, due, first, to changes in recent users' attitudes/perceptions toward smartphone usage monitoring apps, and second, because we upgraded our survey contents when modifying perceived ease of use into EE. In conclusion, the findings of this study comprise an up-to-date empirical study in analyzing factors affecting users' BIU of a smartphone over dependence management monitoring system according to NPSU and PSU.

Similarly, FC→ PE was analyzed to prove that if certain facilitating conditions are met, it would also affect users by making them expect some good performance from the system. Once their expectations of the system's effective performance were high, we predicted that it would naturally link to a positive effect on their intention to use PE→ BIU, and these effects did turn out to be statistically significant. This was congruent with research in many other fields historically (25, 52, 53), which used perceived usefulness (PU) as a similar variable to PE in this research. In 2013, Deng et al. (54) found that perceived value had significant effects on both attitudes toward smartphone health services and BIU. Similarly, Hung et al. (55) found that PU influences BIU because it positively influences users' attitudes toward certain suggested systems. Compared to these studies, the uniqueness of our research lies in the fact that we conducted deeper investigations into some factors like FC, which proved to be the core fundamental before the “PE to BIU” influence relations when adopting e-Health-related systems.

As for EE→ PE, we analyzed whether users' enhanced convenience would positively affect their perception of the system's usefulness. The results supported that lessened EE led to users positively increased PE of the system, meaning that user interface or user experience should be as user-friendly as possible.

The effect of self-regulation on behavioral intentions to use SR→ BIU was not statistically significant. According to a recent related study by van Deursen et al. (3), subjects with high self-regulation demonstrated a willingness to adopt various methods to fight against problematic smartphone use. On the contrary, our proposed research demonstrated that self-regulatory mentality had no significant impact on intentions to use the smartphone usage management app method as a means to intervene in problematic smartphone use.

Lastly, the statistical significance of PS as an important factor in determining users' intention to use the system was valid for both groups under a 95% confidence interval. Personal information, especially in medical fields, is considered to be highly sensitive information that should not be leaked at any cost. The recent findings of this paper are consistent with those of Cimperman et al. (56), who emphasized that PS is one of the three key factors that influence acceptance. Our recent findings were also supported by Ebert et al. (57), who stated that PS significantly affects acceptance of internet-based mental health interventions.

### Difference Between NPSU and PSU

Among the common features discussed above, one hypothesis pathway of the proposed research showed an interesting difference between NPSU and PSU. That is, although the significance of PS as an important factor in determining users' intention to use the system was valid for both groups, the PSU group showed less need for the importance of security than the NPSU group. Related research specifically identifying this issue is extremely rare in the field of smartphone overuse. Similar research by Blachnio et al. (58) regarding the addictive use of the Internet found that Internet addiction was negatively related to PS. This may imply that the PSU group's proneness to addiction somewhat reduced their consciousness for PS. This logical pathway may have caused their statistically significant lower impact of PS on intention to use than the NPSU group.

## Conclusions

This study has investigated the factors affecting users' BIU smartphone usage management apps. Participants were divided into NPSU and PSU groups for an in-depth investigation. Overall, the results showed common features between NPSU and PSU, with facilitating factors positively affecting PE for intentions to use smartphone usage management apps, and with perceived security positively affecting intentions to use smartphone usage management apps. One distinct difference between NPSU and PSU was that the latter attributed a lower importance to perceived security than the former.

A limitation of this research is that the population did not include adolescents, who are known to be heavy smartphone users and particularly susceptible to overusing these devices. Since this study data is from self-assessment information, it can cause recall bias and social satisfaction bias in response.

The results have been used to develop the core risk prediction model embedded in our developed smartphone overuse monitoring system app, which is currently being launched. Post-follow-up future research should be conducted among the served population for further survey investigation. The research results can also be flexibly applied to other medical systems. Some of our highlighted unique points are envisioned to provide intensive insights for broadening knowledge about technology acceptance in the field of e-Addictology (59), and a constant update of research is required to successfully reflect the quickly changing perceptions of adaptive smartphone users.

## Data Availability Statement

The raw data supporting the conclusions of this article will be made available by the authors, without undue reservation.

## Ethics Statement

The studies involving human participants were reviewed and approved by Institutional Review Board of the Catholic University of South Korea, St. Mary's Hospital (MC18QESI0065) approved the study. The patients/participants provided their written informed consent to participate in this study.

## Author Contributions

MC and S-JL: conceptualization, validation, and writing—original draft preparation. MC and MJR: methodology. MC: formal analysis, investigation, writing—review and editing, and visualization. MC and SL: software and data curation. IC: resources, supervision, and project administration. IC and D-JK: funding acquisition. All authors contributed to the article and approved the submitted version.

## Conflict of Interest

The authors declare that the research was conducted in the absence of any commercial or financial relationships that could be construed as a potential conflict of interest.

## Abbreviations

IoT: Internet of Things
IoMT: Internet of Medical Things
App: Application
SOMS: Smartphone Overdependence Management System
TAM: Technology Acceptance Model
ECT: Expectation-Confirmation Theory
FC: Facilitating Conditions
EE: Effort Expectancy
PE: Performance Expectancy
BIU: Behavioral Intention To Use
PS: Perceived Security
SR: Self Regulation
PU: Perceived Usefulness
NPSU: Non-Problematic Smartphone Use
PSU: Problematic Smartphone Use
GFI: Goodness-of-Fit Index
TLI: Tucker–Lewis Index
CFI: Comparative Fit Index
RMSEA: Root Mean Square Error of Approximation
CR: Critical Ratio.
